# Supplementary material for: Risk factors associated with congenital anomalies among newborns in southwestern Ethiopia: A case-control study
Source: PLoS One. 2021 Jan 28;16(1):e0245915. doi: 10.1371/journal.pone.0245915 (PMC7843017; doi:10.1371/journal.pone.0245915)
Supplement: S1 Table — (DOCX) [file pone.0245915.s001.docx]

| **Variables** | **N*** | **Mean** | **Std. Deviation** |
| --- | --- | --- | --- |
| Birth weight (gram) | 1128 | 3028.96±25.075 | 842.155 |
| Gestational age (weeks) | 1135 | 36.42±0.239 | 8.060 |
| Birth order of the infancy | 1123 | 2.43±0.055 | 1.855 |
| Maternal age | 1137 | 25.31±0.151 | 5.083 |
| Paternal age | 1042 | 30.73±0.215 | 6.955 |
| Maternal average monthly income | 639 | 3030±124.430 | 3145.398 |
| Party | 1121 | 1.78±0.088 | 2.959 |
| Gravida | 1120 | 2.46±0.057 | 1.924 |

**S1 Table.** Descriptive analysis (mean and Std. Deviation of maternal and neonatal characteristics of the study participants in relation to birth weight, gestational age, birth order of the infancy, maternal age, paternal age, maternal average monthly income, party and gravida.
