# Supplementary material for: Risk factors associated with congenital anomalies among newborns in southwestern Ethiopia: A case-control study
Source: PLoS One. 2021 Jan 28;16(1):e0245915. doi: 10.1371/journal.pone.0245915 (PMC7843017; doi:10.1371/journal.pone.0245915)
Supplement: S2 Table — (DOCX) [file pone.0245915.s002.docx]

Instead of looking at the 95% CI, logistic regression analyses was repeated by using a narrower interval (e.g. 99% CI) to reduce the significance level from 0.05 to 0.01 (S2 Table). Setting a higher significance threshold for individual comparisons to compensate for the number of inferences was being made. This is one way that we can reduce the probability of getting a false positive.

**S2 Table. Interdependent risk factors adjusted for multiple tests**

| **Variables** | **AOR(95%CI)** |  |
| --- | --- | --- |
| **Antenatal care follow up** | **AOR(99.0% CI)** | **P- value** |
| Not attend at all | 2.952(1.166, 7.472) | .003* |
| 1 to 3 visits | 2.121(1.390, 3.237) | .000* |
| Minimum of 4 visits | 1 |  |
| **Drugs during early pregnancy** |  |  |
| Yes | 3.435(2.012, 5.863) | .000* |
| No | 1 |  |
| **Pesticides** |  |  |
| Yes | 3.926(1.266, 12.176) | .002* |
| No | 1 |  |
| **Passive smoking** |  |  |
| Yes | 4.104(1.892, 8.901) | .000* |
| No | 1 |  |
| **Folic acid supplement** |  |  |
| Yes | 0.639 (0.247, 0.740) | .000* |
| No | 1 |  |
| **History of birth defect** |  |  |
| Yes | 3.741(.875, 16.006) | .019 |
| No |  |  |
| **Water source for drinking** |  |  |
| Pipe | 1 |  |
| Underground water | 1.492(.756, 2.944) | .130 |
| Surface water | 2.073(1.221, 3.519) | 000* |

****Statistically significant (P<0.01)***
