# Supplementary material for: Risk factors associated with congenital anomalies among newborns in southwestern Ethiopia: A case-control study
Source: PLoS One. 2021 Jan 28;16(1):e0245915. doi: 10.1371/journal.pone.0245915 (PMC7843017; doi:10.1371/journal.pone.0245915)
Supplement: S1 Annexes — (DOCX) [file pone.0245915.s003.docx]

**Annexes**

**Annex I: Consent form and questionnaire (English, Amharic and Afaan Oromo Version)**

**ADDIS ABABA UNIVERSITY**

**COLLEGE OF HEALTH SCIENCES**

**SHOOL OF MEDICINE**

**INFORMED CONSENT FORM**

**Investigations of Congenital Anomalies at Birth and Associated Risk Factors in South western Ethiopia**

I, ________________________________ (name), have read the information sheet above and clearly understood the purpose of the research. I have understood all that has been read and had my questions answered satisfactorily. I also understand that I can change my mind at any stage and it will not affect to me in any way. I hereby need to assure with my signature below that I, without any coercion or forceful act by the research team, have decided to voluntarily participate in the study to contribute my part in the effort being made for the investigation of the congenital anomalies at birth and associated risk factors in Southwestern Ethiopia.

**Please** *tick* I **agree to participate/ take part in this research**

**Yes**

**No**

Participant’s signature: Date:

Data collector’s signature: Date:

አዲስአበባዩኒቨርሲቲ

የጤናሳይንስኮሌጅ

የስምምነትፎርም

የአብሮወለድ ጉድለቶችና ተያያዥየ ሆኑ አጋላጭ ምክንያቶች ጥናት በደቡብምዕራብኢትዮጵያ

እኔ __________________________ (ስም) ከላይ የተገለፀውን መረጃ አንብቤያለሁ፡፡ የምርምሩን አላማም በግልፅ ተረድቻለሁ፡፡ ያነበብኩትን በሙሉ የተረዳሁ ሲሆን የነበሩኝጥ ያቄዎችም በሚያረኩኝ መልኩ መልስ አግኝተዋል፡፡ እንዲሁም ምርምሩ እየተካሄደ ባለበት በማንኛውም ጊዜ ሃሳቤን መለወጥ የምችል መሆኑን እና ይህን ማድረጌም ምንም ጉዳት የማያስከትልብኝ መሆኑን ተረድቻለሁ፡፡ በጥናቱ ለመሳተፍም ከምርምር ቡድኑ ምንም አይነት ጫና ያልተደረገብኝ ሲሆን በደቡብም ዕራብ ኢትዮጵያ ለሚካሄደው አብሮወለድ ጉድለቶችን ና ተያያዥ የሆኑ አጋላጭ ምክንያቶችን ለማግኘት በሚደረገው ጥናት የራሴን ድርሻ ለማበርከት በፈቃደኝነት ውሳኔ አድርጌያለሁ፡፡


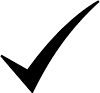
እባክዎ፡ ለመሳተፍ ይህንንም ልክት ያድርጉ

እስማማለሁ

አልስማማም፡ እባክዎ የማይፈልጉ ከሆነ እዚህላይምልክትያድርጉ

የተሳታፊውፊርማ፡______________ ቀን፡________________

የመረጃሰብሳቢውፊርማ፡_______________ ቀን፡________________

**UNIVERSIITII FINFINNEE**

**KOOLEJII SAAYINSII FAYYAA**

WALIIGALTEE HIRMAATOTA WALIINII

**Mata duree**: **Dhibee da’imman waliin dhalatuu fi hiriiroo waantota dhibee kana fidan waliin qaban Kibba Iya Itoopiiyaatii**

Ani, ____________________________ (maqaa), kaniin jedhamu odeefannoo armaan olitti ibsame dubbiseen/naa ibsamee hubadheera. Bu’aa qo’annichaa hubadheen jira. Kana malees gaaffiin gaaffadheef deebii ga’aa arga’een jira. Kanaafuu dirqii tokko malee qoranicha irratti hirmaachuuf walii galeen jira. Qo’annicha irratti hirmaachuukofis mallattooko armaan gadiin mirkaneessuun barbaada.

Hirmaachuukeef sanduqa armaan gadii keessatti mallattoo ‘**√**’ barreessi.

**Ni hirmaata:** **□ Eeyyee!** **□ Lakki**

**Malattoo Hirmaata: ___________________________________ Guyyaa: _______________**

**Maallatoo Daataa Funaana: _____________________________ Guyyaa: ____________**

**Annex 2: Questionnaire**

Addis Ababa University, College Public Health, School Medical Sciences,

Questionnaire format on accessing predisposing risk factors associated to birth defect in southwestern Ethiopia.

**Instructions**

**Part A**_ Information about the neonate: Please fill the question by **mark ‘x’** or **writing the answers**

|  | **Questions** | **Response categories** | **Skip** |
| --- | --- | --- | --- |
| 1 | Date of data collection  (DD/MM/YYYY)  Guyyaa ragaan funaaname  መረጃው የተሰበሰበበት ቀን | ____/____/ ____ |  |
| 2 | Name of health institution  Maqaa dhaabbata fayyaa  የጤና ተቋሙ ስም | ______________________ |  |
| 3 | Card number  Lakkofsa kaardii  የካርድ ቁጥር | ______________________ |  |
| 4 | Date of birth (DD/MM/YYYY)  Guyyaa daa’imni dhalate  ህጻኑ የተወለደበት ቀን | ____/____/ _____ |  |
| 5 | Sex of newborn  Saala daa’ima dhalatee  ልጁ የተወለደበት ቀን | │__│ Male/ Dhiira / ወንድ │__│Female / Dhalaa/ ሰት |  |
| 6 | Birth weight(Gram)  Ulfatina (giraamaan)  የልጁ ክብደት | │_­­­­­­­­_______│ |  |
| 7 | Birth outcome  Bu’aa dhalate  የወሊድ ሁኔታ | Alive birth / fayya dhalte/ በሕይወት የተወለደ  Still birth / du’aa dhalate/ ሙቶ የተወለደ |  |
| 8 | Type birth  Gosa bu’aa dhaloota  የወልድ አይነት | ____Single / qeenxee/ አንድ  --------Twin / lakkuu/ መንታ |  |
| 9 | Gestational age at birth [weeks]  Umurii ulfi itti da’ame (torbaaniin)  የእርግዝና ግዜ በሣምንት | │_­­­­­­­­_______│ |  |
| 10 | Birth order of this infant  Daa’imichi da’ima meeqafadha  ስንተኞ ልጅ ነው | │_­­­­­­­­_______│ |  |
| 11 | Onset of labor  Akkata itti dhufiinsa ciniinsuu  የአወላለድ ሁኔታ | │__│Spontaneous / tasa/በድንገት  │__│Induced/kakkasuu/ማነቃቃት |  |
| 12 | Mode of delivery  Haala da,uumsaa  የወላድ ሁኔታ | │__│Spontaneous Vertex/  │__│Caesarean Section |  |

**Part B_ Mother Characteristics and related factors**

|  | **Questions** | **Response categories** | Skip |
| --- | --- | --- | --- |
| 1 | Age of the mother  Ummurii haadhaa  የእናተ ዕድሜ | │___________│ |  |
| 2 | Maternal Education  Sadarkaa barnoota haadhaa  የትምህርት ደረጃ | │__│Illiterate / kan hin baranne/ ያልተማረ  If literate, grade │_­­­­­­­­_______│ |  |
| 3 | What is your (the mother’s) occupation?  Hojii haadha  የእናተ የሥራ ሁኔታ | │_­­­­­­­­___­­­­­­­­­­­­­­­­­­_______________│ |  |
| 4 | Residential area (Zone/district)  Iddoo jireenyaa  የመኖሪያ አካባቢ | │_­­­­­­­­_______/_­­­­­­­­_______│ |  |
| 5 | Religion  Amantii  ሐይማኖት | │__│Muslim  │__│Orthodox  │__│Protestant  Other specify |  |
| 6 | Source of drinking water:  Madda bishaan dhugaatii  የመጠጥ ዉሀ ሁነታ | │__│Pipe water/ bishan bombaa/የቧንባ ውሃ  │__│Underground water / lafa jala/የጉድጓድ ውሃ  │__│Surface water/bishan lagaa/ የወንዝ ውሃ |  |
| 7 | Parity |  |  |
| 8 | Gravid |  |  |
| 9 | History of abortions  Seenaa ulfa ofirraa baasuu  ከዚህ በፊት ውርጃ ያውቃሉ | │__│Yes /eeyyee/አዎ  │__│No / lakki/ አይደለም |  |
| 10 | History of still births  Seenaa daa’ima du’e da’uu  የሞተ ልጅ ተወልዷል | │__│Yes /eeyyee/አዎ  │__│No / lakki/ አይደለም |  |
| 11 | Pervious history of congenital abnormality  Kanaan dura seenaa “congenital abnormality”  አካል ጉደለት ከዚህ በፊት ተወልዷል | │__│Yes /eeyyee/አዎ  │__│No / lakki/ አይደለም |  |
| 13 | Is there a history of congenital malformation in your family?  Seenaan ‘congenital malformation’ maatii keessaan keessa jiraa?  በቤተሰብ ውሰጥ “congenital formation” ተፈጥሮ ያውቃል | │__│Yes /eeyyee/አዎ  │__│No / lakki/ አይደለም |  |
| 14 | Age of the father  Umurii abbaa  የአባት እድሜ | │____________│ |  |
| 15 | Average monthly income of the family  Giddu gala galii ji’aa maatii  የቤተሰብ ወርሀዊ ገቢ | │_­­­_____│ |  |

**Part C_ The index pregnancy, ask the mother the following questions regarding the index pregnancy (last pregnancy)**

|  | **Questions** | **Response categories** | **Skip** |
| --- | --- | --- | --- |
| 1 | Did you attend antenatal care during the index pregnancy?  Yeroo ulfa kee isa asa ammaa hordoffii da’uumsa duraa gootee turtee?  የወሊድ ክትትል አደረገው ያውቃሉ | │__│Yes /eeyyee/አዎ  │__│No / lakki/ አይደለም |  |
| 2 | If yes, the number visits  Yoo eeyyee ta’e, yeroo meeqa?  አዎ ከሆነ ስንት ጊዜ | │_­­­_____│ |  |
| 3 | Did you use folic acid during the index pregnancy?  Yeroo ulfa kee isa ammaa foolik asiidii fayyadamtee?  በእርግዝና ጊዜ ፎልክ አሲድ ተጠቅመው ያውቃሉ ? | │__│Yes /eeyyee/አዎ  │__│No / lakki/ አይደለም |  |
| 4 | Did you [the mother] take alcohol during pregnancy?  Yeroo ulfa kee alkoolii fayyadamtee beektaa?  በእርግዝና ጊዜ አልኰል ተጠቅመው ያውቃሉ | │__│Yes /eeyyee/አዎ  │__│No / lakki/ አይደለም |  |
| 6 | Did you smoke cigarette during pregnancy?  Yeroo ulfaa tamboo xuuxxee beektaa?  በእርግዝና ጊዜ ሲጋራ ታጫሻለሽ ? | │__│Yes /eeyyee/አዎ  │__│No / lakki/ አይደለም |  |
| 7 | Were you staying with the cigarette smoker during pregnancy, hence passively smoking?  Yeroo ulfa kee namoota tamboo xuuxan waliin turtee?  በእርግዝና ጊዜስ ሲጋራ ከሚያጨሱ ሰዎች ጋር ሁነሽ ታውካለሽ | │__│Yes /eeyyee/አዎ  │__│No / lakki/ አይደለም |  |
| 8 | Were you exposed to X-ray during pregnancy?  Yeroo ulfakee rajii kaatee beektaa?  በእርግዝና ጊዜ X-rey ተነስተው ያውቃሉ | │__│Yes /eeyyee/አዎ  │__│No / lakki/ አይደለም |  |
| 9 | If yes, at what estimated gestation age?  Yoo eeyyee ta’e, yeroo tilmaama ummurii ulfaa ammamitti?  አዎ ከሆነ በስንተኛው እርግዝና ዕድሜዎ | │_­­­_____│ |  |
| 10 | Is there history of exposure to Pesticides? | │__│Yes /eeyyee/አዎ │__│No / lakki/ አይደለም |  |
| 11 | Do you have epilepsy?  Dhukkubaa gagabdoo( lubaabee) qabdaa?  የአዙሪት በሽታ አለቦት ? |  |  |
| 12 | If yes, did you use anti epileptic drugs during pregnancy?  Yoo eeyyee ta’e, yeroo ulfa kee qorichaa farra gagabdoo fayyadamtaa?  አዎ ከሆነ በእርግዝና ጊዜዎ መዳኒቱን ወሰደው ያውቃሉ | │__│Yes /eeyyee/አዎ  │__│No / lakki/ አይደለም |  |
| 13 | Are you diabetes?  Dhukkuba sukaara qabdaa?  የስኳር በሽታ አለቦት | │__│Yes /eeyyee/አዎ  │__│No / lakki/ አይደለም |  |
| 14 | If yes, do use medication regularly  Yoo eeyyee ta’e, qoricha isaa idileen fayyadamtaa?  አዎ ከሆነ መዳሀኒቱ በትክክል ተጠቅመዎል | │__│Yes /lakki/አዎ  │__│No / lakki/ አይደለም |  |
| 15 | Did you have any infections during pregnancy?  Yeroo ulfa kee dhukkubsattee beektaa?  በእርግዝና ጊዜ በበሽታ ተይዘው ያውቃሉ | │__│Yes /eeyyee/አዎ  │__│No / lakki/ አይደለም |  |
| 16 | When was the infection during pregnancy  Yeroon ulfaa dhukkubsatte yoom ture?  በእርግዝና ጊዜሽ አሞሽ ያውቃል | │_­­­_____│ |  |
| 17 | What was it  Yoo eyyee ta’e maal inni  አዎ ከሆነ ምን ዓይነት በሽታ | │_­­­__________│ |  |
| 19 | Did you use antibiotics?  Qorichaa farra baakteeriyaa fayyadamtee?  ፀረ-ባክተሪያ መዳሃኒት ውስደው ያውቃሉ | │__│Yes /eeyyee/አዎ  │__│No / lakki/ አይደለም |  |
| 20 | Did you [the mother] take ARVs during pregnancy?  Yeroo ulfaa qoricha farra HIV fudhatee beektaa?  በእርግዝና ጊዜ ፀረ HIV መዳሀኒት ውስደው ያውቃሉ | │__│Yes /eeyyee/አዎ  │__│No / lakki/ አይደለም |  |
| 21 | If yes in above, mention the type/types of ARVs taken  Yoo eeyyee ta’e gosa isaa ibsi?  አዎ ከሆነ ዓይነቱን ይንገሩን |  |  |
| 22 | For how long did you take ARVs?  Qorichaa farra HIV kana hammamiif fudhate?  ፀረ HIV መዳሀኒት ለምን ያህል ጊዜ |  |  |
| 23 | Do you have asthma?  Dhukkuba asmii qabdaa  አስም አለቦት | │__│Yes /eeyyee/አዎ  │__│No / lakki/ አይደለም |  |
| 24 | Did you take a drug during the first three months of the pregnancy?  Yeroo ulfa kee ji’oota sadan duraaf qoricha fudhattee beektaa?  በመጀመሪያ ሶስት ወራት መዳሀኒት ወስደው ያውቃሉ | │__│Yes /eeyyee/አዎ  │__│No / lakki/ አይደለም |  |
| 25 | If **yes**, please specify the name of the drug(s)  Yoo eeyyee ta’e, qorichicha nuuf ibsitaa?  አዎ ከሆነ ምን ዓይነት መዳሀኒት ነው | │_­­­___________│ |  |
| 26 | Did you take a drug between the 4^th^ and 6^th^ months the pregnancy?  Ji’a ulfa kee 4^ffaa^ fi 6^ffaa^ gidduutti qoricha fudhatee beektaa?  በአራተኛው እርግዝና ወራት ውስጥ መዳኒት ወስደው ያውቃሉ | │__│Yes /eeyyee/አዎ  │__│No / lakki/ አይደለም |  |
| 27 | If **yes**, please specify the name of the drug(s)  Yoo eeyyee ta’e, qorrichichaa nu ibsi  አዎ ከሆነ የመዳኒቱን ዓይነት ገለፁልን | │_­­­___________│ |  |
| 28 | Did you take a drug(s) during the last three months of the pregnancy?  Ji’oota sadan dhuma yeroo ulfa keetti qoricha fudgattee beektaa?  በመጨረሻው ሶስት የዕርግዝና ወራት ውስጥ መዳኒት ወስደው ያውቃሉ? | │__│**Yes** /eeyyee/አዎ  │__│**No** / lakki/ አይደለም |  |
| 29 | If **yes**, please specify drug(s)  Yoo eeyyee ta’e, qorichicha nuu ibsi  አዎ ከሆነ የመዳኒቱን ዓይነት ይንገሩን | │_­­­___­­­­­________│ |  |
| 30 | Did you drink coffee during pregnancy  Yeroo ulfaketti buna dhugaa turtee?  ቡና በዕርግዝና ጊዜ ይጠጡ ነበረ | │__│Yes /eeyyee/አዎ  │__│Nolakki/ አይደለም |  |
| 31 | Did you chew khat during pregnancy?  Yeroo ulfaketti caatii qaamaa turtee?  ጫት በዕርግዝና ጊዜ ይቅሙ ነበረ | │__│Yes /eeyyee/አዎ  │__│No / lakki/ አይደለም |  |
| 32 | Do you have hypertension disorder?  Dhibee dhiibaa dhiigaa qabdaa?  የደም ግፊት በሽታ አለቦዎት | │__│Yes /eeyyee/አዎ  │__│No/ lakki/ አይደለም |  |

**ART D: Information about the presence and types of congenital anomalies**

| Congenital malformation | | |
| --- | --- | --- |
| **Are there congenital anomalies** | │__│ Yes │__│ No |  |
| **If yes, what types of birth defects or anomalies does the child have? Mark ‘x’** | | |
| **Central Nervous system**   1. │__│ Hydrocephalus 2. │__│ Meningomyelocele 3. │__│ Microcephaly 4. │__│ Anencephaly 5. │__│ Encenphocele 6. │__│ Craniorachischisis | | |
| **Gastrointestinal defects**   1. │__│Trachea-esophageal fistula 2. │__│gastroschisis 3. │__│Diaphragmatic hernia 4. │__│Duodenal atresia 5. │__│Imperforate anus 6. │__│Ectopic anus 7. │__│Congenital inguinal hernia 8. │__│Umbilical hernia 9. │__│Epigastric hernia | | |
| **Genitourinary defects**   1. │__│Amibigious genitalia 2. │__│Urethral fustula 3. │__│Meatal stenosis 4. │__│Penile tourniquet 5. │__│Hypospadias 6. │__│Epispadias | | |
| **Musculoskeletal defects**   1. │__│Spinal bifida 2. │__│Club foot: bilateral _________ unilateral 3. │__│Ankyloglossia 4. │__│Palatine fistula 5. │__│Clefet lip only: median ____,bilateral_____, unilateral___ 6. │__│Cleft palate only: median ____,bilateral_____, unilateral___ 7. │__│Cleft lip with palate: median ____,bilateral_____, unilateral___ 8. │__│Fistula in the neck 9. │__│Congenital torticilis 10. Others:___________________________________ | | |

**Data Collector**

1. **Name: _____________________________**
2. **Date: ___________________________________**

**Signature: _______________________________**
