# Supplementary material for: Risk factors associated with congenital anomalies among newborns in southwestern Ethiopia: A case-control study
Source: PLoS One. 2021 Jan 28;16(1):e0245915. doi: 10.1371/journal.pone.0245915 (PMC7843017; doi:10.1371/journal.pone.0245915)
Supplement: Attachment — Submitted filename: Reponse to Reviewers.docx [file pone.0245915.s006.docx]

**Date: November 21, 2020**

**A Point-by-Point Response to Academic Editor and Reviewer(s)**

**Journal: PLOS One**

**MS ID:PONE-20-23170**

**Dear editor,**

**We thank you for your invaluable comments, suggestions and recomendations in our submission of the revised manuscript: We included all as per comments and responses are represented in bold**

- A rebuttal letter that responds to each point raised by the academic editor and reviewer(s). You should upload this letter as a separate file labeled ‘Response to Reviewers.’
- **Comment accepted. The rebuttal letter that responds to each point raised by the academic editor and reviewer(s) is included. The letter is uploaded as a separate file and labeled ‘Response to Reviewers’**
- A marked-up copy of your manuscript that highlights changes made to the original version. You should upload this as a separate file labeled **‘**Revised Manuscript with Track Changes’
- **Comment accepted. The changes made to the original version is highlighted in orange color in the revised manuscript. The file is labeled as Revised Manuscript with Track Changes’.**
- **Dear editor,**

**The orange higlighted in result part of the abstract and in an independent predictors of CAs of the result part including in the Table 6 and 7 were not because of changes made to the original result but simply to indicate that we removed the P – value as per your recommendations.**

- An unmarked version of your revised paper without tracked changes. You should upload this as a separate file labeled ’‘**Manuscript**'.
- **Comment accepted. The unmarked version of the revised paper without tracked changes labeled as ‘Manuscript’**
- If you would like to make changes to your financial disclosure, please include your updated statement in your cover letter. Guidelines for resubmitting your figure files are available below the reviewer comments at the end of this letter.
- **The financial disclosure and updated statement included inthe cover letter**

PLOS ONE

Journal Requirements:When submitting your revision, we need you to address these additional requirements.

1. Please ensure that your manuscript meets PLOS ONE’s style requirements, including those for file naming.

**Dear editor,**

- **We thank you for reminding us to check our manuscript in lines with PLOS ONE's style requirements. We checked journal formats and corrected it accordingly.**

2. Please include additional information regarding the checklist and questionnaire used in the study and ensure that you have provided sufficient details that others could replicate the analyses.

For instance, if you developed a questionnaire and/or checklist as part of this study and it is not under copyright more restrictive than CC-BY, please include a copy, in both the original language and English, as Supporting Information.

**Dear editor,**

- **We uploaded all required ducuments as supporting informations including questionnaire written inthe English, Afaan Oromo, and Amharic languages as supporting information.**

3. Thank you for stating the following in the Competing Interests section:

‘The authors, Mr. Soressa Abebe, and Dr. Girmai Gebru receive a salary from Addis Ababa University, Dr. DemisewAmanu, Professor Zeleke Mekonnen, and Mr. Lemessa Dube receivea salary from Jimma University.The authors have declared that no competing interests exist.’

a. Please confirm that this does not alter your adherence to all PLOS ONE policies on sharing data and materials, by including the following statement: “This does not alter our adherence to  PLOS ONE policies on sharing data and materials"."(as detailed online in our guide for authors [http://journals.plos.org/plosone/s/competing-interests](about:blank)).  If there are restrictions on sharing of data and/or materials, please state these. Please note that we cannot proceed with consideration of your article until this information has been declared.

- **We confirm this does not alter our adherence to PLOS ONE policies on sharing data and materials.**

**Funding**

**This study obtained fundings from Addis Ababa University and Jimma University for data collection. The funding Universities do not have any role in the study design, data collection, analysis and interpretation of data and writing of the manuscript.**

 b. Please include your updated Competing Interests statement in your cover letter; we will change the online submission form on your behalf.

- **We included the updated competing interest statement in our cover letter.**
- **The authors have declared that no competing interests exits**

Please know it is PLOS ONE policy for corresponding authors to declare, on behalf of all authors, all potential competing interests for the purposes of transparency. PLOS defines a competing interest as anything that interferes with, or could reasonably be perceived as interfering with, the full and objective presentation, peer review, editorial decision-making, or publication of research or non-research articles submitted to one of the journals. Competing interests can be financial or non-financial, professional, or personal. Competing interests can arise in relationship to an organization or another person. Please follow this link to our website for more details on competing interests: [http://journals.plos.org/plosone/s/competing-interests](about:blank)

- **The corresponding author declared on behalf of all authors**

4. PLOS requires an ORCID iD for the corresponding author in Editorial Manager on papers submitted after December 6th, 2016. Please ensure that you have an ORCID iD and that it is validated in Editorial Manager. To do this, go to 'Update my 'Information'(in the upper left-hand corner of the main menu), and click on the Fetch/Validate link next to the ORCID field. This will take you to the ORCID site and allow you to create a new iD or authenticate a pre-existing iD in Editorial Manager. Please see the following video for instructions on linking an ORCID iD to your Editorial Manager account: <https://www.youtube.com/watch?v=_xcclfuvtxQ>

- **The corresponding author linked his ORCID iD**

5. Please amend your list of authors on the manuscript to ensure that each author is linked to an affiliation. 'Authors'affiliations should reflect the institution where the work was done (if authors moved subsequently, you can also list the new affiliation stating ""current affiliation":…."as necessary).

- **We included the updated list of authors and their corresponding affiliation as per request.**

Reviewers' comments:

**Comments to the Author**

1. Is the manuscript technically sound, and do the data support the conclusions?

The manuscript must describe a technically sound piece of scientific research with data that supports the conclusions. Experiments must have been conducted rigorously, with appropriate controls, replication, and sample sizes. The conclusions must be drawn appropriately based on the data presented.

Reviewer #1: Partly

2. Has the statistical analysis been performed appropriately and rigorously?

Reviewer #1: No

3. Have the authors made all data underlying the findings in their manuscript fully available?

The [PLOS Data policy](http://www.plosone.org/static/policies.action#sharing) requires authors to make all data underlying the findings described in their manuscript fully available without restriction, with rare exceptions (please refer to the Data Availability Statement in the manuscript PDF file). The data should be provided as part of the manuscript or its supporting information, or deposited to a public repository. For example, in addition to summary statistics, the data points behind means, medians and variance measures should be available. If there are restrictions on publicly sharing data—e.g. participant privacy or use of data from a third party—those must be specified.

Reviewer #1: No

4. Is the manuscript presented in an intelligible fashion and written in standard English?

PLOS ONE does not copyedit accepted manuscripts, so the language in submitted articles must be clear, correct, and unambiguous. Any typographical or grammatical errors should be corrected at revision, so please note any specific errors here.

Reviewer #1: Yes

5. Review Comments to the author

Please use the space provided to explain your answers to the questions above. You may also include additional comments for the author, including concerns about dual publication, research ethics, or publication ethics. (Please upload your review as an attachment if it exceeds 20,000 characters)

**Point –by- point response to the reviewer**

**Dear reviewer,**

**We thank you for your invaluable comments, suggestions and recommendations. We have amended the manuscript based on your constructive comments as follows:**

Reviewer #1: Major comments:

This article is a retrospective study on the association between maternal exposure to risk factors during early pregnancy and the development of congenital anomalies. The study was conducted based on data collected from six selected hospitals in southwestern Ethiopia through two years. The data analysis provided exciting data and analysis on the possible correlation between possible risk factors and congenital anomalies.

One issue with the experimental design is the sampling method to select the six hospitals. The authors mentioned that the hospitals were selected purposively based on their capacity to carry out the required assessment. This non-randomness of sampling may sharply limit the generalization of the conclusion to newborns in the whole area. This point has been mentioned in the “Limitation of the Studysection. But without providing more evidence that these hospitals can represent all the hospitals in the area, the authors should be more careful when they tried to generalize the conclusions.

- **We thank you for raising this critical comment; although we mentioned purposive selection of the hospitals, we have included all hospitals except one. So, we feel it can be generalizable to the whole study area. Besides our indication of the limitations, we have now updated the revised manuscript indicating that almost all hospitals six out of seven in the study area were assessed.**

One major issue with the statistical analyses is that the author conducted multiple tests during their studies, while they used a threshold P-value of 0.05 for significance, which is a typical cut-off when doing a single test. I would suggest that they use an adjusted p-value instead to control the false positive rate for this multiple test case.

- **Thank you again for your constructive comment. We just considered P-value for candidate selection. We used the 95% CI interval for the final decision to minimize complications/false positive rate. If the confidence interval includes a null value, we declared not statistically significant.**

Also, it would be more informative if a formula for the final regression model with corresponding coefficients was provided beside the descriptions.

- **Thank you for the comment. We Now included the final regression formula as per your invaluable recommendation as follows.**

$$\log\left( \boldsymbol{odds of CA} \right)\boldsymbol{=-1.997+1.234}\left( \boldsymbol{drug use} \right)\boldsymbol{+1.368}\left( \boldsymbol{pesticide exposure} \right)\boldsymbol{+1.412}\left( \boldsymbol{passive smoker} \right)\boldsymbol{-0.358}\left( \boldsymbol{folic acid} \right)\boldsymbol{+1.349 (history of CA+0.729}\left( \boldsymbol{surface water} \right)\boldsymbol{+1.082}\left( \boldsymbol{no atenatal care visits} \right)\boldsymbol{+0.752 (1 to 3 antenatal care visit)}$$

Also, there should be a separate section where data availability is described. The authors should show where the original questionnaire and raw data can be found for their readers.

- **Thanks for the suggestion about the availability of data and materials. We have attached the requieed documents as supporting information with the revised manuscript.**

**Availability of data and materials**

- All relevant data are within the manuscript and its supporting information

Minor comments:
Table 7: It would be clearer if the variable name is in a separate column from the variable levels.

- **Re: We thank you for your constructive comment. We have corrected.**

 Regards!

Soressa Abebe
