# Supplementary material for: Risk factors associated with congenital anomalies among newborns in southwestern Ethiopia: A case-control study
Source: PLoS One. 2021 Jan 28;16(1):e0245915. doi: 10.1371/journal.pone.0245915 (PMC7843017; doi:10.1371/journal.pone.0245915)
Supplement: Attachment — Submitted filename: Response to Reviewers.docx [file pone.0245915.s007.docx]

**Date: January 7, 2021**

**A Point-by-Point Response to Academic Editor and Reviewer(s)**

**Journal: PLOS One**

**MS ID: PONE-D-20-23170R1**

**MS title:**Risk factors associated with congenital anomalies among newborns in southwestern Ethiopia: A case-control study

**Dear editor,**

**We thank you for your invaluable comments, suggestions and recommendations in our revised manuscript,**

**Now we included responses and corrections as per your comments and suggestions for each point raised either by the editor or the reviewer’s as follows**

- A rebuttal letter that responds to each point raised by the academic editor and reviewer(s). You should upload this letter as a separate file labeled 'Response to Reviewers'.
- ***The rebuttal letter is prepared and uploaded as a separate file and labeled ‘Response to Reviewers’***
- A marked-up copy of your manuscript that highlights changes made to the original version. You should upload this as a separate file labeled 'Revised Manuscript with Track Changes'
- ***The changes made to the original version are highlighted in orange color in the revised manuscript. The file is labeled as ‘Revised Manuscript with Track Changes’.***
- An unmarked version of your revised paper without tracked changes. You should upload this as a separate file labeled 'Manuscript'.
- ***The unmarked version of the revised paper without tracked changes labeled as ‘Manuscript’***
- If you would like to make changes to your financial disclosure, please include your updated statement in your cover letter. Guidelines for resubmitting your figure files are available below the reviewer comments at the end of this letter.
- **No changes made**

Reviewers' comments:

Reviewer's Responses to Questions

**Comments to the Author**

1. If the authors have adequately addressed your comments raised in a previous round of review and you feel that this manuscript is now acceptable for publication, you may indicate that here to bypass the “Comments to the Author” section, enter your conflict of interest statement in the “Confidential to Editor” section, and submit your "Accept" recommendation.

Reviewer #1: All comments have been addressed

2. Is the manuscript technically sound, and do the data support the conclusions?

The manuscript must describe a technically sound piece of scientific research with data that supports the conclusions. Experiments must have been conducted rigorously, with appropriate controls, replication, and sample sizes. The conclusions must be drawn appropriately based on the data presented.

Reviewer #1: Yes

3. Has the statistical analysis been performed appropriately and rigorously?

Reviewer #1: N/A

4. Have the authors made all data underlying the findings in their manuscript fully available?

The [PLOS Data policy](http://www.plosone.org/static/policies.action#sharing) requires authors to make all data underlying the findings described in their manuscript fully available without restriction, with rare exception (please refer to the Data Availability Statement in the manuscript PDF file). The data should be provided as part of the manuscript or its supporting information, or deposited to a public repository. For example, in addition to summary statistics, the data points behind means, medians and variance measures should be available. If there are restrictions on publicly sharing data—e.g. participant privacy or use of data from a third party—those must be specified.

Reviewer #1: Yes

5. Is the manuscript presented in an intelligible fashion and written in Standard English?

PLOS ONE does not copyedit accepted manuscripts, so the language in submitted articles must be clear, correct, and unambiguous. Any typographical or grammatical errors should be corrected at revision, so please note any specific errors here.

Reviewer #1: Yes

6. Review Comments to the Author

Please use the space provided to explain your answers to the questions above. You may also include additional comments for the author, including concerns about dual publication, research ethics, or publication ethics. (Please upload your review as an attachment if it exceeds 20,000 characters)

**Reviewer #1:** The modifications are reasonable to all comments except the one for the multiple test issue. The multiple test issue would not be solved by replacing p-values with significance intervals. If a 95% CI is used, then significance level is still 5%. The authors should also adjust their significance levels and choose a more restrict CI to adjust for the multiple test issue.

**Comment accepted:**

**Dear reviewer, we thank you for raising this critical comment. It is acceptable that instead of looking at the 95% CI, logistic regression analyses was repeated by using a narrower interval (e.g. 99% CI) to reduce the significance level from 0.05 to 0.01. Setting a higher significance threshold for individual comparisons to compensate for the number of inferences was being made. This is one way that we can reduce the probability of getting a false positive.Therefore, as per your invaluable comment, we corrected and adjusted the multiple tests to 99% confidence interval and significance level of 0.01(1%) and indicated it in the revised manuscript in (table 6 and 7) as well as in the text under independent predicator (risk factors) of congenital anomalies.**

**Interdependent risk factors adjusted for multiple tests are indicated and uploaded as supporting information as S2 Table for your considerations.**

Best regards!

Soressa Abebe
